# Assessment of the effects of dexmedetomidine on outcomes of traumatic brain injury using propensity score analysis

**DOI:** 10.1186/s12871-022-01822-2

**Published:** 2022-09-02

**Authors:** Jinbu Xu, Qing Xiao

**Affiliations:** 1 Critical Care Medicine Department, Foshan Women and Children Hospital, No. 11, Western Renmin Road, Foshan City, 528000 Guangdong Province China; 2 Critical Care Medicine Department, Lianyungang Second People’s Hospital, No. 161, Xingfu Road, Lianyungang City, 222000 Jiangsu Province China

**Keywords:** Dexmedetomidine, Traumatic brain injury, Survival analysis, Propensity score analysis

## Abstract

**Background:**

Dexmedetomidine was found to be protective against traumatic brain injury (TBI) in animal studies and safe for use in previous clinical studies, but whether it improves TBI patient survival remains to be determined. We sought to answer this question by analyzing data from the MIMIC clinical database.

**Methods:**

Data for TBI patients from the MIMIC III and MIMIC IV databases were extracted and divided into a dexmedetomidine group and a control group. In the former group, dexmedetomidine was used for sedation, while in the latter, it was not used. Parameters including patient age, the Acute Physiology score III, the Glasgow Coma Scale, other sedatives used, and pupillary response within 24 h were employed in propensity score matching to achieve a balance between groups for further analysis. In-hospital survival and 6-month survival were analyzed by Kaplan–Meier survival analysis and compared by log-rank test. Cox regression was used repeatedly for the univariate analysis, the multivariate analysis, the propensity score-matched analysis, and the inverse probability of treatment weighted analysis of survival data. Meanwhile, the influences of hypotension, bradycardia, infection, and seizure on outcome were also analyzed.

**Results:**

Different types of survival analyses demonstrated the same trend. Dexmedetomidine significantly improved TBI patient survival. It caused no more incidents of hypotension, infection, and seizure. Hypotension was not correlated with in-hospital mortality, but was significantly correlated with 6-month mortality.

**Conclusions:**

Dexmedetomidine may improve the survival of TBI patients. It should be used with careful avoidance of hypotension.

**Supplementary Information:**

The online version contains supplementary material available at 10.1186/s12871-022-01822-2.

## Background

Traumatic brain injury (TBI) is a critical public health problem worldwide. Globally, approximately 69 million individuals suffer TBI from all causes each year [1]. TBI leads to disability and death, and places a substantial socioeconomic burden on every country. Therefore, guidelines based on clinical research were designed by different medical communities and associations to provide high-quality care to TBI victims and improve their outcomes [2–4]. Among the strategies included in these guidelines, sedatives and analgesics was recommended to reduce intracranial pressure (ICP) and the cerebral metabolic rate of oxygen (CMRO2), to control seizures and facilitate compatible mechanical ventilation. Dexmedetomidine (Dex), with its unique characteristics of sedation without respiratory depression and residual metabolites, concomitant analgesic and sympatholytic effects, and no interference in neurological assessment or weaning from mechanical ventilation, was presumably considered suitable for the sedation of TBI patients. However, although many basic studies have suggested Dex’s neuroprotective effects in TBI patients, the available clinical evidence is insufficient to prove its benefits on TBI outcomes [5–7].

This study was designed to assess the effects of Dex on the survival of TBI patients, and to demonstrate that despite its side effects, Dex is still a sedative that helps improve TBI patients’ prognosis.

## Methods

### Study design

We conducted a retrospective cohort study based on the MIMIC III and MIMIC IV databases, which are large, freely-available databases comprising de-identified health-related data from patients admitted to the critical care units of the Beth Israel Deaconess Medical Center. The MIMIC III database contains data for 58,976 ICU admissions between 2001 and 2012, and the MIMIC IV database contains data for 524,520 admissions between 2008 and 2019 [8, 9]. The data were extracted by the certified author Jinbu Xu (certificate number: 25508977). The study complied with the RECORD guideline for reporting items specific to observational studies using routinely collected health data [10].

### Data acquisition

Patients aged between 14 and 100 years, with one of the following diagnoses, were included: traumatic brain injury, intracranial injury, and skull fracture with loss of consciousness. For patients who were admitted to the hospital several times, only the first admission data were collected. Those who stayed in ICU for less than 24 h were excluded. The patients were grouped into the Dex group and the control group. In the Dex group, Dex was infused intravenously, while in the control group, no Dex was used. The clinical characteristics collected included the following: age, sex, acute physiology score (APS) III, Charlson Comorbidity Index (CCI) score, the Glasgow Coma Scale (GCS) score, whether sedatives other than Dex were used (other sedatives that were considered included midazolam, propofol, and ketamine, with 0 for used, and 1 for never used), and pupillary response within 24 h after admission (graded into three levels: reactive to light (RL), one eye nonreactive to light (ONRL), both eyes nonreactive to light (BNRL). The variables of interest were extracted from the MIMIC III and MIMIC IV databases using Navicat 15 for PostgreSQL and codes from MIMIC Code Repository (https://github.com/MIT-LCP/mimic-code). The variables were chosen according to clinical experiences and literature [11–14]. If the variables were measured repeatedly within 24 h of admission, the worst values were chosen. Outcomes included: in-hospital survival (living state upon hospital discharge, alive or dead), 6-months survival (living state 180 days after admission). In counting in-hospital survival rates, hospital length of stay (LOS) was employed and calculated as days from ICU admission to hospital discharge. Those who stayed in ICU for more than 54 days were recorded as “alive” at that time point. To further assess the confounding factors on outcomes, data on hypotension, infection, and seizure in TBI patients were collected and analyzed. The hypotension data were recorded as the percentage of mean arterial pressure (MAP) under 65 mmHg. The infection and seizure data were recorded as dichotomous data.

### Sample size estimation

A sample size estimation was calculated using the survival analysis in Power Analysis and Sample Size software (PASS 15). The overall in-hospital mortality of TBI patients was reported to be around 12.3%, and we expect an increase in survival rate by 8% in the Dex group [15]. The following settings were used: power = 0.8, alpha = 0.05, Group Allocation = Equal (N1 = N2), and alternative hypothesis = two-sided test, therefore the least numbers of measurement required were *N* = 173 for each group.

### Statistical analysis

Quantitative variables with a normal distribution are expressed as the mean and standard deviation, and those with a skewed distribution are expressed as the median and interquartile range (Q1, Q3). Qualitative variables were expressed in percentages. If the proportion of patients with missing data was less than 5%, the data were removed; otherwise, they were multiply imputed.

Cox proportional-hazards regression was performed to estimate the association between Dex use and survival. Initially, an univariate analysis of linkage between Dex use and outcome was performed. Then a multivariable analysis was performed using covariates that include age, gender, the APS III score, the CCI score, the GCS score, other sedative usage, and pupillary response. Then, to achieve a better balance between the groups and avoid selection bias, propensity score matching (PSM) and propensity score-based inverse probability of treatment weighting (IPTW) were used to adjust the covariates [16]. Variable selection from the previously collected variables was made using stepwise backward method using the Akaike information criterion (AIC). In the PSM analysis, propensity scores were estimated by multivariate logistic regression analysis of the selected clinical characteristics. Nearest neighbor matching without replacement (1:1), with a caliper setting of 0.05, between the groups was performed using the R package “Matching”. In the IPTW analysis, the estimated probabilities from the propensity-score model were used to calculate the inverse probability of treatment weights. Standardized mean differences (SMDs) were calculated to assess the covariate-balancing efficacy of PSM and IPTW, and to examine the strong ignorability of treatment assignment assumption. Subsequently, Cox models were established for the propensity score-matched data and the inverse-probability-weighted data, and hazard ratios (HRs) with 95% conference intervals (CIs) calculated. A by-group survival analysis was visualized by Kaplan–Meier curves. Statistical analyses were performed in R studio (R version 4.2.0). A value of *p* < 0.05 (two-tailed) was considered statistically significant.

The incidence of hypotension, infection and seizure were compared between groups. Skewed distribution data were assessed by the rank sum test. The counting data were tested using the chi-square test or the Fisher’s exact test. Fisher’s Exact test was used if the theoretical frequency was less than five.

Sensitivity analysis was carried out by constructing a logistic regression model using the original unmatched and matched data with confounding factors added. The linkage between Dex usage and patient mortality (in-hospital mortality and 6-month mortality) was reassessed, and the influences of hypotension, infection, and seizure on outcome were evaluated in IPTW adjusted data.

## Results

### Baseline characteristics

**Fig. 1:**
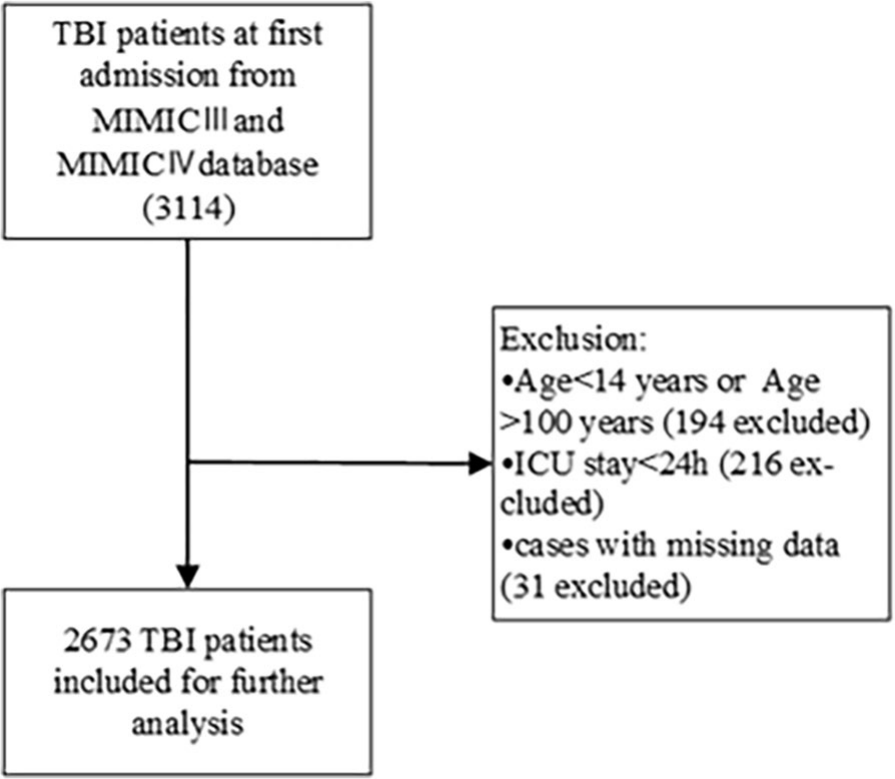
Study Flow Diagram in the Present Study

**Fig. 2:**
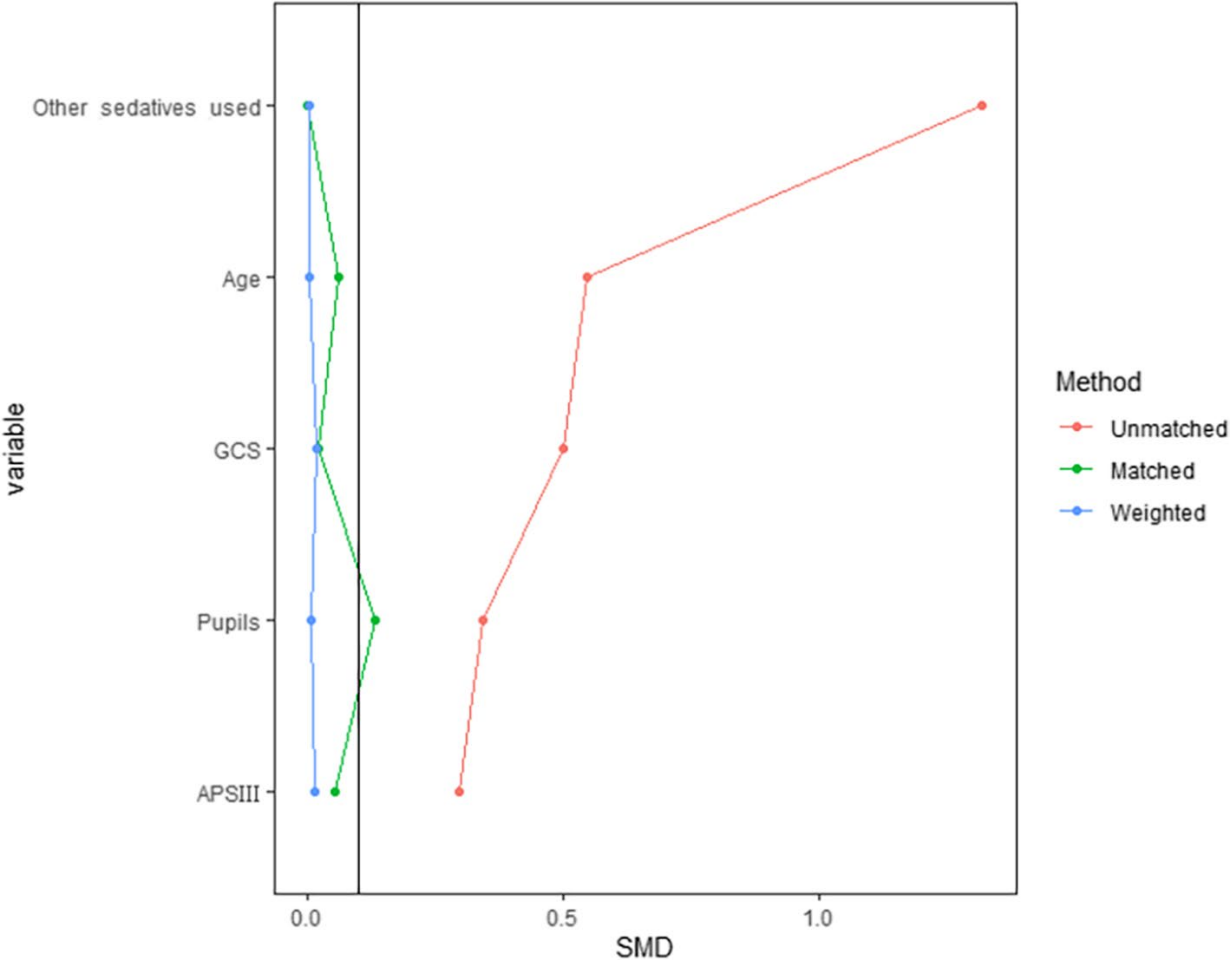
Standardized mean difference (SMD) of variables before and after propensity score matching and weighting. The unmatched data showed SMDs far beyond 0.1 in age, the APS III score, the GCS score, pupillary response. The propensity score matching (PSM) and inverted probability of Treatment weighting (IPTW) significantly reduce SMDs to less than 0.1, with IPTW achieved the best effect

Of the 3114 initially admitted TBI patients, of 441 patients were excluded. A total of 194 patients were excluded because they did not meet the age criteria. Another 216 patients were excluded because they stayed in the ICU for less than 24 h. Thirty-one patients with missing values for the collected clinical characteristics were also excluded. Thus, 2673 TBI patients were included for further analysis (Fig. 1). Among the patients, 175 were included in the Dex group, and 2498 were included in the control group. All the variables, including age, APS III score, the GCS score, other sedatives used, and pupillary response, were considered to assess the balance between the groups. The SMDs of all the variables between groups fall far outside 0.1, which indicates a significant imbalance (Table 1). Propensity score matching and propensity score-based IPTW significantly improved the imbalance, with IPTW resulting in the best effect. The sample size after propensity score matching was 175 for each group. (Fig. 2, Supplement material: Table S1 and S2).

**Table 1:** Baseline Characteristics of TBI Patients

Characteristics	Control (*n* = 2498)	Dex (*n* = 175)	SMD
Age, Median (Q1, Q3)	59.25 (38.92, 77.5)	43 (27, 61.35)	0.546
Female (%)	907 (36)	40 (23)	0.298
APS III, Median (Q1, Q3)	33 (25, 45)	42 (32, 53)	0.295
CCI, Median (Q1, Q3)	3(1, 5)	1 (0, 4)	0.404
GCS, Median (Q1, Q3)	13 (9, 14)	9 (7, 12.5)	0.499
Other Sedatives used(%)	1226 (49)	171 (98)	1.318
Pupils (%)			0.341
BNRL	213 (9)	31 (18)	
ONRL	57 (2)	10 (6)	
RL	2228 (89)	134 (77)	

*control* The control group, *Dex* The dexmedetomidine group, *APS III* Acute Physiology Score III, *CCI* Charlson Comorbidity Index, *GCS* Glasgow Coma Scale, *BNRL* Both Eyes Nonreactive to Light, *ONRL* One Eye nonreactive to Light, *RL* Reactive to Light

### Outcomes

**Fig. 3:**
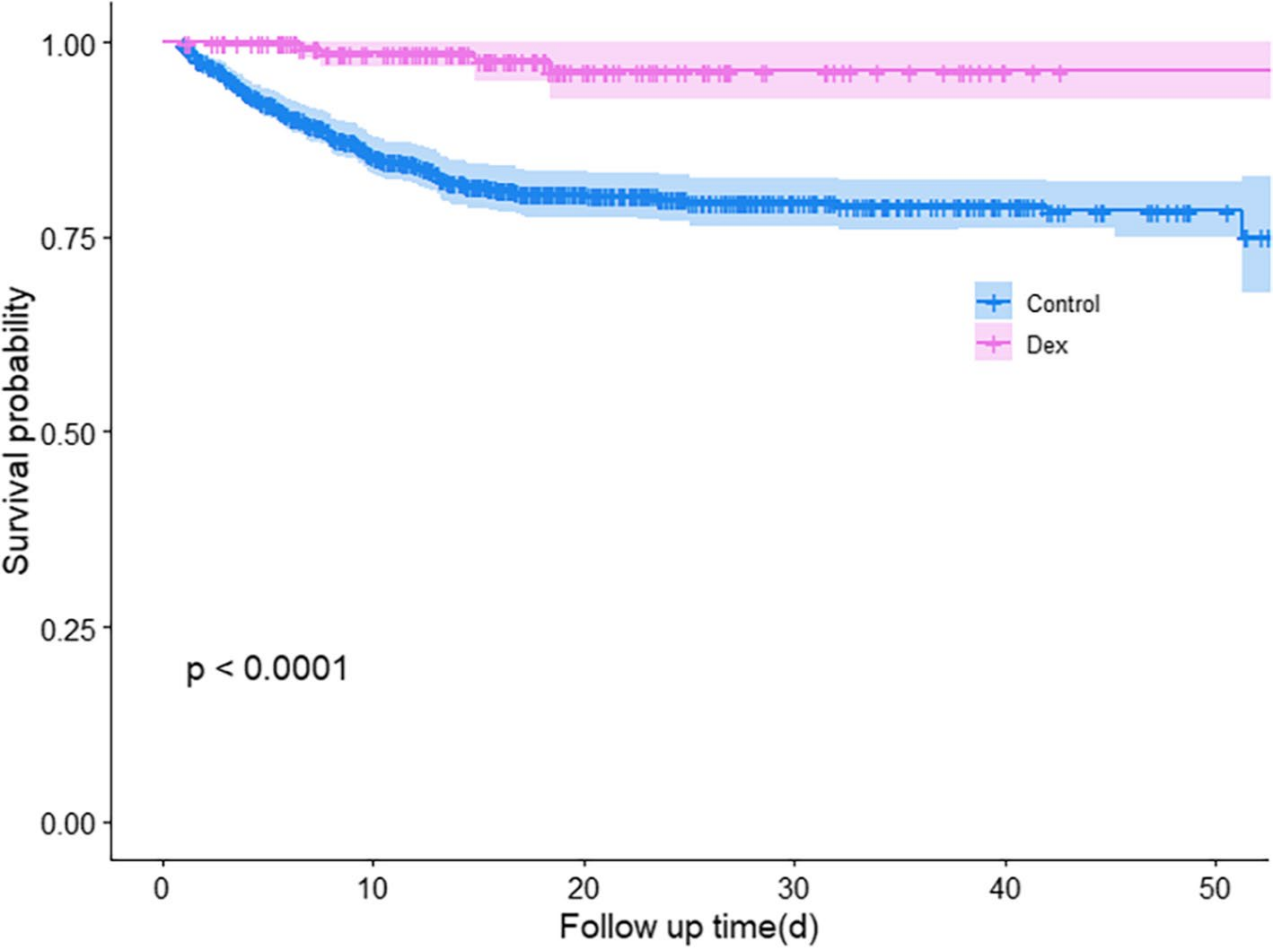
Post-IPTW In-hospital Survival Analysis. The Kaplan–Meier curves based on IPTW analysis showed that upon hospital discharge the Dex group showed a significant better survival. Those stayed in hospital for longer than 54 days was taken as survival. Control the control group, Dex the dexmedetomidine group

Upon discharge, 2.3% of the TBI patients in the Dex group died, while in the control group, 11.6% of them lost their lives. The unadjusted univariate analysis of Dex usage and outcome showed that the patients in the Dex group were less likely to die during the hospital stay than those in the control group (hazard ratio, 0.12; 95% CI, 0.04 to 0.31). The unadjusted multivariable analysis showed a hazard ratio of 0.13 and 95% CIs of 0.05–0.35. Cox regression based on propensity score-matched data showed a hazard ratio of 0.16 and 95% CIs of 0.06–0.47. Cox regression based on IPTW-adjusted data showed a hazard ratio of 0.12 and 95% CIs of 0.05 to 0.32. These results all suggest similar life-saving results for Dex (Table 2). The Kaplan–Meier curves based on IPTW analysis showed that Dex significantly improved the hospital survival of TBI patients (*P* < 0.001, Fig. 3).

**Table 2:** Association between Dex Use and in-hospital survival in the Crude Analysis, Multi variable Analysis, and Propensity Score Analysis

Analysis	Death
No. of death/no. of patients (%)	
Dex	4/175 (2.3)
control	290/2498 (11.6)
Univariable analysis—hazard ratio(95% CI)	0.12 (0.04- 0.31)
Multivariable analysis—hazard ratio(95% CI)	0.13 (0.05- 0.35)
Propensity-score analyses—hazard ratio(95% CI)	
With matching	0.16 (0.06- 0.47)
With inverse probability weighting	0.12 (0.05- 0.32)

*Control* The control group, *Dex* The dexmedetomidine group

**Fig. 4:**
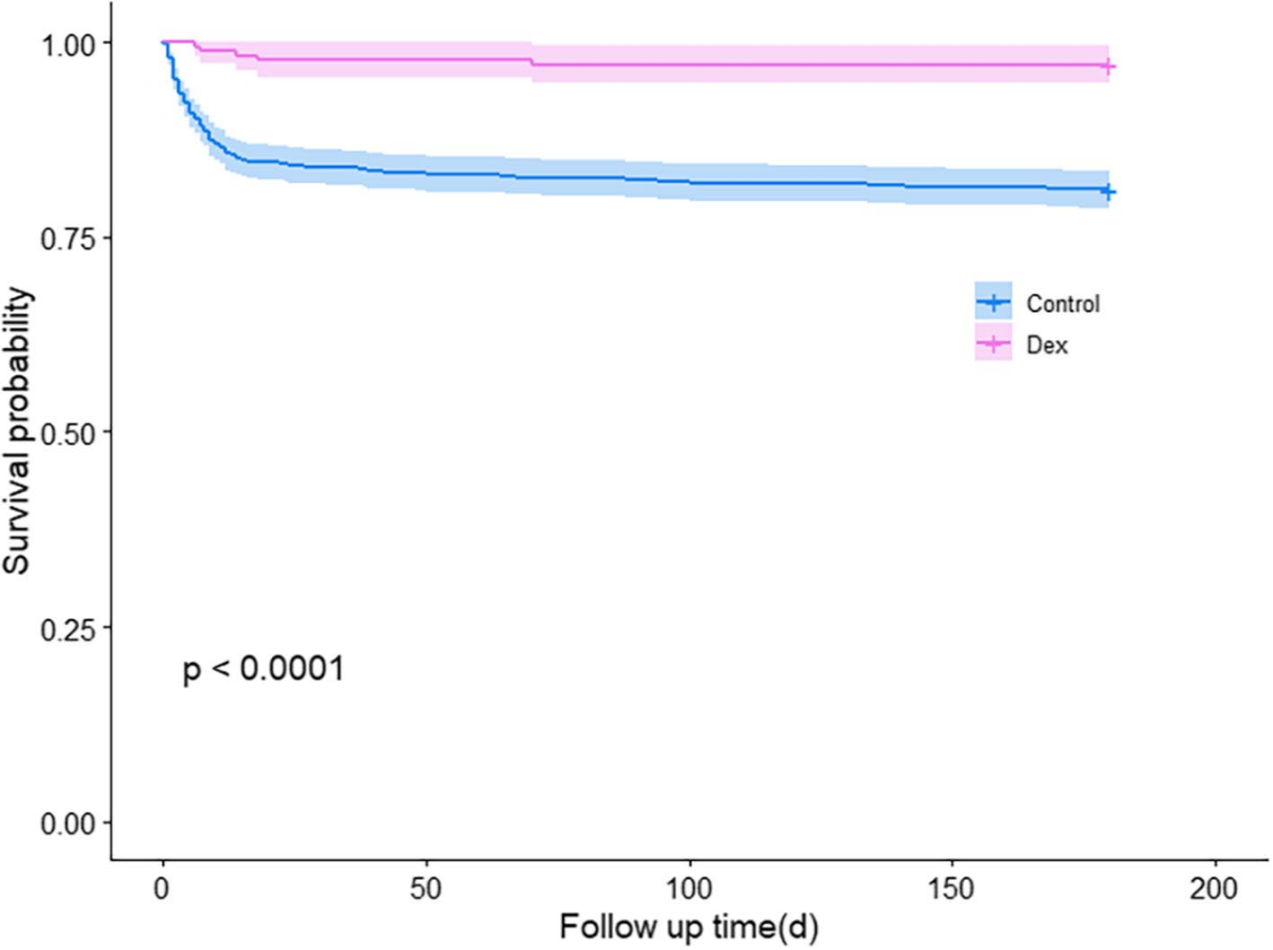
Post-IPTW 6-month Survival Analysis. The Kaplan–Meier curves based on IPTW analysis showed that 6 months after admission the Dex group showed a significant better survival. Control the control group, Dex the dexmedetomidine group

Six months after hospital admission, 2.9% of the TBI patients in the Dex group died, while in the control group, 17.7% of them lost their lives. The unadjusted univariate analysis, the unadjusted multivariable analysis, the analysis based on propensity score-matched data, and the analysis based on IPTW-adjusted data all showed that the Dex group was less likely to die, with the hazard ratio and 95% CIs to be 0.15 (0.06- 0.36), 0.14 (0.06- 0.35), 0.13 (0.05- 0.33), 0.14 (0.06- 0.33), respectively (Table 3). The Kaplan–Meier curves based on IPTW analysis showed that Dex significantly improved the 6-month survival of TBI patients (*P* < 0.001, Fig. 4).

**Table 3:** Association between Dex Use and 6-month survival in the Crude Analysis, Multivariable Analysis, and Propensity Score Analysis

Analysis	Death
No. of death/no. of patients(%)	
Dex	5/170 (2.9)
control	442/2498 (17.7)
Univariable analysis—hazard ratio(95% CI)	0.15 (0.06- 0.36)
Multivariable analysis—hazard ratio(95% CI)	0.14 (0.06- 0.35)
Propensity-score analyses—hazard ratio(95% CI)	
With matching	0.13 (0.05- 0.33)
With inverse probability weighting	0.14 (0.06- 0.33)

*Control* The control group, *Dex* The dexmedetomidine group

### Complications

Unmatched Data on complications showed no statistical differences between the Dex group and the control group in the incidence of hypotension. The occurrence of infection and seizure was significantly higher in the Dex group. Propensity score-matched data showed no significant differences in the incidence of hypotension, infection, and seizure (Table S3).

### Sensitivity analysis

The logistic regression on IPTW adjusted data showed that Dex significantly correlated to patient outcome, whether in-hospital mortality or 6-month mortality. The factor of infection and seizure showed no significant correlation to patient outcome. Hypotension was found not correlated to in-hospital mortality, but positively correlated to 6-month mortality (Table 4).

**Table 4:** Multivariate logistic regression conducted for association between adverse reactions in-hospital mortality and 6-month mortality on IPTW adjusted data

	**OR**	**CI**	***P* value**
**in-hospital mortality**			
Hypotension	9.59	0.50 ~ 142.32	0.107
Infection	0.56	0.16 ~ 1.53	0.300
Seizure	0.74	0.00 ~ 11.08	0.876
Dex	0.14	0.04 ~ 0.36	< 0.001^a^
**6-month mortality**			
Hypotension	15.40	1.12 ~ 200.55	0.036
Infection	0.64	0.23 ~ 1.56	0.358
Seizure	0.56	0.00 ~ 8.35	0.765
Dex	0.12	0.04 ~ 0.30	< 0.001^a^

^a^Significantly associated to outcome at 0.05 level

## Discussion

Our study demonstrated that Dex significantly improved the survival of TBI patients. It is by and large a sound effect, whether by univariate Cox regression analysis or multivariate analysis. The propensity score-matched and the IPTW adjusted data revealed the same trend. Regarding the TBI complication, hypotension was not significantly higher in the Dex group than that in the control group in both unmatched and propensity score-matched data. The Dex group showed significantly higher incidences of infection and seizure in unmatched data, but showed no statistical difference between groups after the data were matched. Logistic regression showed infection and seizure were not significant risk factors for both in-hospital and 6-month mortality. Hypotension, though was not shown to be a significant risk factor for in-hospital mortality, was shown to be a significant factor for 6-month mortality. These suggest that hypotension is an adverse reaction to be aware of, but can be avoided or reduced when used in selected patients. Logistic regression also showed Dex usage was the only independent protective factor for patient outcome.

Although many studies have previously examined the effects of Dex on TBI, none of them ascertained the survival-facilitating effect of Dex in the clinical context. Studies carried out in murine TBI models suggested Dex’s protective effects. Wu et al. showed that Dex prevented the injured brain from tissue lesions and cell death, and reduced axonal injury and synaptic degeneration if used at a dose of 100 µg/kg [6]. Other studies demonstrated that Dex exerts its protective effects through anti-inflammatory properties via suppression of NF-κB and NLRP3 inflammasome activation through the attenuation of endoplasmic reticulum stress-induced apoptosis [5, 17, 18]. Kara-kaya et al. further proved that different doses of Dex all attenuated neuroinflammation [19]. However, although Dex had long been considered “promising” in the “Lund concept” put forward by Lund University, Sweden, clinical investigators were cautious in validating its survival-facilitating effect [20]. In 2013, a study suggested that Dex can be used in TBI patients without affecting brain oxygenation [21]. In 2016, another study demonstrated that Dex infusion in TBI patients does not worsen neurological functioning [22]. Recently, Dex was found to be associated with a reduction in paroxysmal sympathetic hyperactivity and agitation in TBI patients [23, 24]. Although opposite opinions exist considering Dex’s side effects of reducing blood pressure and heart rate, researchers agreed that more studies would be necessary to evaluate Dex’s effects on TBI patients.

In the present study, the limited number of patients who used Dex can be explained in clinical practice. In ICU practice, there are several choices for sedation. Those most frequently used for TBI include propofol, midazolam, ketamine, and Dex [20]. As Dex is not the only choice, and it has not been confirmed to be beneficial to TBI patients, it is not used as widely as some of the other sedatives. In our study, the use of other sedatives was balanced between groups using propensity score analysis.

Other baseline characteristics included in the analysis (age, gender, the APS III score, the CCI score, the GCS score, and pupillary response) are parameters that are usually considered in prognosis judgement [11, 12]. APS III and CCI scores were calculated to reflect the disease severity and chronic health status, respectively. They are substitutes for the APACHE III score, which also includes an APS III part and a chronic health status part. Although fewer items on chronic health status are included in the APACHE III scoring system, they could not be wholly collected from the MIMIC database. The CCI contains more items (17) on chronic health status, which can be easily obtained [25]. It is common for TBI patients to have other combined injuries. Still, these injuries must cause organ dysfunction or occur in frail people to result in mortality, which can be evaluated by the APS III and CCI. Nevertheless, after variable selection with AIC, only age, the APS III score, the GCS score, other sedatives used, and the pupillary response were retained for further propensity score matching or weighting. With the propensity score matching and weighting method, the imbalance of baseline characteristics was basically corrected, for the SMD of the covariates between groups were controlled within 0.1 at large. This would make the following regression analysis conclusions more tenable.

Although the present study answered the question of whether Dex improves the survival of TBI patients, there are still limitations. The first is that the study included only patient survival data but not long-term neurological recovery data, which was not collected in the MIMIC III and MIMIC IV databases. Second, a limited number of TBI patients in this study received dexmedetomidine, so it was difficult to perform more fine-grained subgroup analyses. For example, the types of brain injury may play their roles on patient outcomes but cannot be further divided and balanced in our study. As a result, it is difficult to differentiate the effect of TBI subtypes, such as epidural hematoma and subdural hematoma, midline shift, or basal cistern compression on the outcome. A similar case is the other sedatives used. Other sedatives used in TBI patients can be further classified into propofol, midazolam and ketamine subgroups. The three sedatives might have their influence on outcomes, which cannot be further analyzed. Third, unmeasured confounders may exist and have influences, which may discount the robustness of our conclusion. In short, future studies are warranted to enroll more TBI patients using Dex and to consider more relevant details.

## Conclusions

In conclusion, the results of this study warrant the use of Dex in TBI patients. It may improve the survival of TBI patients, and brings no apparent adverse reaction of hypotension, infection, or seizure. Hypotension may influence 6-month mortality, so it is advisable to keep aware of it in using Dex. Nevertheless, large-scale clinical trials are needed to confirm our results.

## Supplementary Information

**Additional file 1: Table S1.** Characteristics of propensity score-matched data. **Table S2.** Characteristics of inverse probability-weighted data. **Table S3.** Incidence of hypotension, occurrence of infection and seizure in unmatched data and propensity score matched data.

## Data Availability

The datasets used and analyzed during the current study are available from the author Jinbu Xu upon Email to 598278274@qq.com.

## Abbreviations

AIC: Akaike information criterion
APS: Acute physiology score
BNRL: Both eyes nonreactive to light
CCI: Charlson Comorbidity Index
CIs: Conference intervals
CMRO2: Cerebral metabolic rate of oxygen
Dex: Dexmedetomidine
GCS: Glasgow Coma Scale
HRs: Hazard ratios
ICP: Intracranial pressure
IPTW: Inverse probability of treatment weighting
LOS: Length of hospital stay
ONRL: One eye nonreactive to light
PSM: Propensity score matching
RL: Reactive to light
SMD: Standardized mean differences
TBI: Traumatic brain injury
