# Supplementary material for: Assessment of the effects of dexmedetomidine on outcomes of traumatic brain injury using propensity score analysis
Source: BMC Anesthesiol. 2022 Sep 2;22:280. doi: 10.1186/s12871-022-01822-2 (PMC9438148; doi:10.1186/s12871-022-01822-2)
Supplement: Supplementary file 1 — Additional file 1: Table S1. Characteristics of propensity score-matched data. Table S2. Characteristics of inverse probability-weighted data. Table S3. Incidence of hypotension, occurrence of infection and seizure in unmatched data and propensity score matched data. [file 12871_2022_1822_MOESM1_ESM.docx]

Table S1 Characteristics of propensity score-matched data

| Characteristics | Control  (n=175) | Dex  (n=175) | SMD |
| --- | --- | --- | --- |
| Age (mean (SD))  APS Ⅲ (mean (SD))  GCS (mean (SD))  Other sedatives used (mean (SD))  Pupils (%)  BNRL  ONRL  RL | 46.75 (21.36)  42.39 (22.19)  9.64 (4.10)  0.98 (0.15)  29 (16.6)  16 (9.1)  130 (74.3) | 45.52 (20.00)  43.48 (18.59)  9.56 (3.31)  0.98 (0.15)  31 (17.7)  10 (5.7)  134 (76.6) | 0.060  0.053  0.021  <0.001  0.035  0.132 |

Abbreviations: Control the control group, Dex the dexmedetomidine group, APS Ⅲ the Acute Physiology Score Ⅲ, GCS Glasgow Coma Scale, BNRL Both Eyes Nonreactive to Light, ONRL One Eye nonreactive to Light, RL Reactive to Light

Table S2 Characteristics of inverse probability-weighted data

| Characteristics | Control  (n=175.44) | Dex  (n=175.00) | SMD |
| --- | --- | --- | --- |
| Age (mean (SD))  APS Ⅲ (mean (SD))  GCS (mean (SD)) Other sedatives used (mean (SD))  pupils (%)  BNRL  ONRL  RL | 45.56 (21.24)  43.72 (23.05)  9.50 (4.21)  0.98 (0.15)  31.1 (17.8)  10.2 (5.8)  134.1 (76.4) | 45.52 (20.00)  43.48 (18.59)  9.56 (3.31)  0.98 (0.15)  31.0 (17.7)  10.0 (5.7)  134.0 (76.6) | 0.002  0.011  0.017  0.001  0.004 |

Abbreviations: Control the control group; Dex the dexmedetomidine group, APS Ⅲ Acute Physiology Score Ⅲ, GCS Glasgow Coma Scale, BNRL Both Eyes Nonreactive to Light, ONRL One Eye nonreactive to Light, RL Reactive to Light

Table S3. Incidence of hypotension, occurrence of infection and seizure in unmatched data and propensity score matched data

| Control Dex P value |
| --- |
| Hypotension (%)  Unmatched 4(1-11) 0 (0-14) 0.562  PSM 3(1-11) 4(1-11) 0.221  Infection (yes/no)  Unmatched 407/2091 43/132^*^ 0.006  PSM 32/143 43/132 0.193  Seizure (yes/no)  Unmatched 25/2473 6/169^*^ 0.013  PSM 3/172 6/169 0.502 |

Data are expressed as median (interquatile range)

Abbreviations: Control the control group; Dex the dexmedetomidine group; PSM propensity score matched.

**^*^** P<0.05 compared to the Control group.
